# Annotations

**Published:** 1897-01-09

**Authors:** 


### ANNOTATIONS.

### Filth and Epidemics.

NOTHING is more stimulating to sanitary progres than the occurrence of great epidemics. So long as Death levies his daily tax in various coins, and kills by means of different diseases, no one cares; but directly any one disease claims an unusual number of victims, then people tremble and sanitation has its way. To the epidemics of cholera which England suffered from between 1831 and 1866, we owe much of the improved water supply and general sanitation of our great towns. To the recent outbreaks of plague Hong Kong owes a great sanitary awakening; and now it is the turn of Bombay. But what we would especially draw attention to is the fact that although people are dying of plague in large numbers in that city, the frightful mortality which is going on there is not due to the plague alone, but to a rise in the prevalence of the ordinary filth diseases which are common in that city. Taking the middle of last month, according to the *Times of India* for December 18th, the mortality had risen to 1,310 per week, which was 840 in excess of the mean of the corresponding weeks in the preceding five years. But of these only 259 died from plague, and it cannot be too strongly insisted on that the plague, which is causing all the terror, is but a final climax to an already alarming death-rate, and that the diseases which produce this wasteful loss of life are the known results of filth and insanitation. If the published sanitary returns are to be trusted, "remittent fever" is killing more people every day even than plague; but remittent fever depends largely on defective cleanliness and on fouling of the soil. Again, in regard to phthisis, which we are every year associating more strictly with insanitary conditions, we find in Bombay a high ordinary death-rate, which has lately increased enormously. A phthisis death-rate of 11 per 1,000, at any time of the year, is something monstrous. It is insanitation, not plague alone, which is the real trouble in Bombay. The annual death-rate in London from all causes is about 18 per 1,000. There is a district in Bombay, named Kammatteepoora, where in recent weeks the death-rate has reached the alarming figure of170·94 per 1,000!

### Sugar as a Food.

THAT the average Briton is a beef-eating and beer-drinking individual seems to be generally accepted. It would seem, however, that of late years the versatile faculty possessed by the Anglo-Saxon race of appropriating for its own purposes all good things has shown itself in a new direction, for in addition to being the great meat eaters, the English-speaking races are now the great sugar eaters of the world. In an interesting article which appears in this month's *Science Progress* it is stated that we eat more than three times as much sugar per head as the Germans do, although it is in Germany that the great mass of the sugar is produced. The fact is that we in these small isles are appropriating nearly half the European consumption of sugar, and nearly a quarter of the world's supply. The question arises at once whether this great glut of sugar is good for the race; whether it is a mere case of luxurious spice eating; or whether sugar is really a food and a source of muscular energy. So far as the use of the raw juice of the cane is concerned the universal experience of the inhabitants of the West Indies is to the effect that, notwithstanding the exposure and the hard work entailed in the reaping of the sugar crop, both men and beasts improve in appearance, in health, and, above all, in muscular power as the harvest proceeds, in consequence of the fact that during that time they simply live upon the sugar-cane and its expressed juices. It may be objected, and with perfect correctness, that the raw juice of the sugar-cane is very different from prepared sugar. No doubt sugar pure and simple is not likely to be so wholesome, or to be capable of being used advantageously in such quantities, as when it is taken direct from the cane and eaten as a fruit. On the other hand, it is unquestionable that sugar is a force producing food. In a paper read before the Royal Society in 1893, Dr. Vaughan Harley described experiments made by him on the subject, the results of which went to show that sugar taken alone on a fasting day increased his capacity for work; that the addition of sugar to normal diet had a marked effect in retarding fatigue; and that by taking $8\frac{3}{4}$ oz. of sugar, in addition to the ordinary diet, the work of an eight hours' day was increased 22 to 36 per cent. We have, then, no reason to be ashamed of our liking for sugar, and it is not impossible that there may be some not altogether remote connection between our national sugar bill and our athletic sports. In view of the opinion which has lately gained ground to the effect that the actions and cravings of early childhood represent the remains of instinct, it is worth bearing in mind how fond children are of sweet things—a trace, perhaps, of an old instinct in the choice of proper food.

### Two Millions for Science.

THE late Mr. Alfred Nobel has left about two millions sterling, which is to be divided into five equal portions for the endowment of science. The third of these portions is to be devoted to the furtherance of physiology and medicine, and the fourth is for the most distinguished literary contribution in the same field. It is to this part of the testator's will that we wish to call special attention at the present moment. Science, more especially physiological and medical science, suffers enormously for lack of able exposition. The inventor, the man who has a "good thing" for sale, often fails to realise a fortune because he has not the means, or does not know the best methods of "placing his goods upon the market." In like manner, discoveries are made in science from time to time, improvements are made in the art of healing, which remain a dead letter to the world, sometimes for years, and sometimes permanently, because there has been no man of adequate expository faculty to place them in clear, intelligent, interesting, and convincing terms before all those who have actual or potential interest in them. As a case in point, chloroform may be mentioned. Sir James Simpson had not the merit of discovering chloroform, nor even of making the first experiments with it. He had the merit of so convincing the medical mind that its general use became a necessity with hardly a day's undue delay. But the real discoverer was a medical student whose name is unknown to most of the medical men of our own time Science which is not adequately expounded is lost to the world, and Mr. Alfred Nobel has shown a rare practicality in devoting so much as one-fifth of a large fortune to the furtherance of science exposition.

